# Microsurgical technique in obstetric brachial plexus repair: a personal experience in 200 cases over 10 years

**DOI:** 10.1186/1749-7221-2-1

**Published:** 2007-01-10

**Authors:** Jörg Bahm, Claudia Ocampo-Pavez, Hassan Noaman

**Affiliations:** 1 Euregio Reconstructive Microsurgery Unit, Franziskushospital Aachen, Germany

## Abstract

We present our personal operative technique in exposing and repairing obstetric brachial plexus (obp) lesions. This technical description of the operative procedure and the strategic choice for the neurotisations are analysed with special regards on the follow-up of these patients (always performed by the surgeon), the histological quality of the proximal root stumps used for cable grafting, and the general reconstruction principles established in international workshops.

We would like to encourage debate on these detailed considerations wherever they could affect the functional outcome.

## Background

There are a lot of scientific contributions related to obp lesions, either general or focused on specific aspects of diagnosis and treatment [1].

Few of them deal with surgical details about nerve exposure and reconstruction and their possible influence on the outcome [2-5].

After 10 years of regular surgical practice on brachial plexus lesions in children and adults, it seems to me that technical aspects and details should be described and discussed more often, although this expertise wount fulfill criteria of EBM (evidence based medicine), but rather reflect surgical practice and its attempt to improve functional results for the paralysed limb [1].

### Indication for surgery, patient's age

According to an agreement among most of the international experts, we operate non or poorly recovering plexus lesions **early **– the total lesion with a flail hand and a Horner sign at 3 months; the upper lesion between 6 and 9 months – this delay just to appreciate the quality of reinnervation in the proximal nerves and muscle targets (shoulder and biceps muscles): when recovery in an upper lesion is very poor, we operate earlier (between 3 and 6 months), when the recovery seems promising and continous, we add time up to the 9^th ^month.

Our decision for surgical exposure and reconstruction is only based on clinical arguments – although we are aware that electrophysiologic criteria like in Birch's group [6], where a continous co-operation between the surgeon and the neurologist exists, are extremily worthfull in the decision-making for upper lesions with various recovery.

### Preoperative imaging

Children born with a breech presentation and showing a severe upper obp lesion often present avulsions of the upper C5 and C6 roots. Although the indication for surgery is evident, preoperative MRI may give further evidence of root avulsion (unvisible radicellae on thin transverse sections, presence of meningoceles).

As our patients come from all over Germany with individual preoperative assessment, we have to share different qualities of both imaging technique and expertise in interpretation. The risk of false negative results is high – where the myelon seems intact and the avulsion is found intraoperatively.

Centers with interdisciplinary brachial plexus teams (like our neighbours in Heerlen and Leiden, The Netherlands) certainly would better integrate the preoperative imaging (neurosurgeons often perform themselves myelography just before starting plexus exploration), although these informations are not decisive for establishing the surgical indication.

The time schedule proposed for the plexus exploration might be non respected if the child is ill just before the scheduled day of surgery (upper airway infection) or if it presents late on the first consultation. Occasionally, we explored children with severe upper lesions even 18 and 21 months old, when the proximal recovery in the shoulder was very bad and biceps activity absent.

### Exposure

The child is supine, the head turned to the contralateral side, no pillow. We always use a single transverse supraclavicular incision, about 4 cm long, 1 cm above the clavicle in total lesions and about 2 cm above in upper lesions.

Once subcutaneous tissue and platysma has been sectioned, we dissect a large quadrangular space limited by the clavicle beneath, the internal jugular vein medially, the emergence of the phrenic nerve from C5 upward, and the upper trunk laterally. The fat-lymphatic tissue bulk is reflected laterally and the upper trunk identified exiting under the scalenus anterior muscle.

### Dissection and neurolysis

The phrenic nerve is identified on the anterior scalenus muscle, stimulated, and followed more proximally to identify the contribution from C5 and the sensitive branches of the cervical plexus.

Than, the trunks and roots down to their foramen are identified progressively and put on rubber loops.

In our country, most of the upper and total lesions are within the supraclavicular space and can be exposed completely by the unique supraclavicular transverse incision, as the soft tissue is elastic and might be spread easily by a self-retaining distractor. When an extended infraclavicular approach is necessary, a vertical delto-pectoral incision may be added and the clavicle might be isolated on a loop, to identify a retroclavicular lesion or adhesions.

We spend enough time to follow all the roots onto the foramen, analyse the scalenus muscle shape (we actively search for the presence of anatomic variants like a scalenus minimus muscle or suprapleural bands) and underlying bone prominences (hypertrophied transverse process of 7^th ^vertebra, between the roots C7 and C8, or a cervical rib).

The emergence of the long thoracic nerve exiting from C5 and C6 very proximally must be identified and spared; as the nerve than enters the middle scalenus muscle, it is more protected.

Distally, normally at the level of the upper clavicular rim, we identify the branches of the upper (and sometimes middle trunk) and especially the suprascapular nerve (SSC). We appreciate if this nerve has been delocalised from its initial topography; sometimes it appears under the clavicle – which is a stigma of a severe traction injury.

If there is a need for proper exposure or grafting, the clavicle is osteotomized in about 10%; we prepare 4 drill wholes to synthetize it by a 3/0 Maxon suture immediately after the grafting.

Once the lesion appears clearly and is delimitated, we perform an intraoperative electrostimulation.

### Electrical stimulation of roots and distal branches

Actually, we only use a stimulation device (Stimuplex^® ^DIG, Braun Melsungen AG, Germany), delivering direct current (monophasic, rectangular, duration 0,1 msec, frequency 1 or 2 Hz) between 0–5 mA (by steps of 0,01 mA – but used at 0,1 mA for our purposes) through a Contiplex^® ^needle (1.2 × 45 mm length), normally used by our anaesthesiologists to perform peripheral nerve blocks, to identify muscle targets and the conductiveness of neuromas. We don't have experience with SSEP or NCV measurements.

Only recently, we added in isolated cases a multichannel neurologic monitoring (Eclipse by ANT software bv, Jodoigne Belgium), especially to study SSEP.

We then perform a drawing of the lesion and have a talk to the parents outside the OR, explaining the exact amount of lesion, the reconstructive plan and possible outcome.

Meanwhile, the sural grafts may be taken and probes of the proximal and distal coaptation sites are sent for specialised neuropathological examination.

### Graft harvest

The sural nerve(s) are harvested through 4 or 5 short oblique skin incisions. No pull is exerted on the nerve. The nerve is kept within a moist swab, until conditioning occurs just before the grafting. The skin is closed by single inverted dermal stitches and elastic Steristrips are applied.

### Neuropathology

When the decision of neuroma resection has been made, we continue dissection of the rootlets onto the foramen and we resect at a level of macroscopic healthy tissue. To prepare the grafting, we want to assure good proximal and distal nerve stump quality.

Each root sample is marked by ink on the proximal side and sent on a moist swab for immediate neuropathological examination. The distal nerve (trunk or cord level) might be examined more easily under magnification; here histologic samples are not taken routinely from all targets, but only from major trunks or connections with doubtful quality.

The examination takes about 30 minutes, after a car transfer to the Institute of Neuropathology (Professor Dr J. Weis) at Aachen University, lasting for another 15 minutes.

We get the results when the grafts have been harvested, normally around noon.

The quality of samples is described by the fascicular pattern (number and orientation of nerve fascicles, presence of peri- or endoneural fibrosis), remnants of nerve degeneration (clusters of Schwann cells called „Büngner" bands), indirect signs of reinnervation (presence of minifascicles, level of myelinisation), and the absence of ganglion cells.

Using this type of macro-(through the surgeon's magnification loop while dissecting) and micro-(through the neuropathologist's microscopic view) confrontation, we try to improve our quality of root microdissection.

We prepare a good fascicular surface to receive sural graft stumps and we look actively for any argument related to more proximal root damage (intraforaminal rupture, partial or total root avulsion) to prevent a grafting on rather worthless stumps.

A root specimen taken too distal on an avulsed root would show normal nerve fibers, without ganglion cells, and thereby the neuropathologist would qualify this a good root – so the level, skill and experience of root dissection is mandatory.

The surgical benefit of this histopathological examination results in the increased attention to the coaptation partners; a risk might emerge if the histologic quality is quoted without analysing other arguments like absence of local neuroma formation (this could indicate a partial avulsion), rapid and complete denervation of targets (without any electric reactivity), or a very proximal lesion (with high risk of centripedal neuron death and lack of regeneration anyway).

We have tried to correlate root quality and clinical outcome in every patient follow-up; and there seems to be a strong concordance, but we still doubt on issues where a rather good stump e.g. put on the inferior trunk gave a insufficient motor recovery of finger flexors.

We also recognise that so far, we don't have a valuable staining tool to distinguish motor from sensitive fascicles or areas, and that topographic arrangement within a root might be random in small roots, with less predictable outcomes (although the topographic description by Millesi et al. [7] is a wonderful work, needing further consideration and refinement).

### Reconstruction, coaptation

As a principle, we distinguish the strategy in total lesions from the (extended) upper lesions.

### Total lesion

According to Gilbert [4], we consider the hand to be the priority in (sub)total lesions. The root Th1 seems to deliver the major motor contribution to the hand – therefore in a total lesion with a Horner sign we always graft on the contribution from Th1 to the inferior trunk.

We also would graft on a distal C8 contribution to enhance hand function in a setting without Horner sign where a basic grip is powered by a good Th1 root and C8 seems damaged.

The other functions are supplied depending on available roots. Priorities are the biceps and shoulder function, than the radial nerve. The long thoracic nerve is often spared.

In general, we favor intraplexic neurotisation on the remaining 2 or 3 proximal roots. C6 seems to be the best motor donor, so we reserve it for the upper trunk; C7 is suitable for the hand function. C5 is often very small, it might neurotise the middle trunk – but this last structure might even be neglected in very severe cases where proximal donors are lacking.

Indication for primary intercostal nerve neurotisation is very rare (3 cases, put on the motor musculocutaneus nerve) and a contralateral C7 transfer (on the median nerve) only has been performed once.

There might be debate if the suprascapular nerve in these total lesions (where medial rotation of the shoulder will be weak) should be neurotised by the distal branch of the accessory nerve; as this nerve might be spared to keep the trapezius muscle intact and to provide a good motor donor for secondary free gracilis muscle transfers.

### Upper lesion

Biceps and triceps activity easily might become co-activated, so we always separate grafts and roots for the upper and middle trunk (resp. the musculocutaneous and radial nerve area).

The shoulder (the axillary nerve and pectoral muscle reinnervation) needs a major motor input and we regularly attribute this role to the best motor root, believed to be C6.

For the suprascapular nerve in upper obp lesions, we strongly advocate extraplexic neurotisation by the distal branch of the spinal accessory nerve, as we need a counterbalance to the medial rotators, as rotational dysbalance at the shoulder level with increasing glenohumeral dysplasia is a frequent issue [8].

A graft from C5 onto the SSC nerve is not reliable to provide enough motor power to the infraspinatus muscle, as the topographic arrangement might not be matched reliably.

Some neurotisations of the SSC nerve give bad results; this might reflect a very distal lesion to this nerve close to the scapula, within the narrow supraspinal notch. This is a place rather difficult to explore surgically, as the spinati muscle must be removed out of their fossae to see the motor nerves entering the muscles from behind – this is a much too agressive procedure, but in cases with concomittant connatal glenohumeral subluxation, where direct trauma to the shoulder girdle is obvious, the hypothesis of a distal lesion of the SSC nerve should be debated.

We spend enough time to prepare the grafts (2 to 4 cm long), resecting the ends, orientating them in an antidromic way and giving enough length to intercalate them as a preformed bundle without tension between good quality proximal and distal targets, proofed by neuropathological examination.

The coaptation is performed fascicle per fascicle under magnification 5 fold or the microscope.

We prefer fibrin glue (Tissucol) when there is no tension; sometimes adaptive stitches are mandatory before the glue. As in severe proximal lesions, the root tissue might be cut back onto/into the foramen, only glue fixation can be applied on these sites.

After all connections have been completed, and the clavicle synthetized, the soft tissue paddle is put over the grafted brachial plexus. Skin closure is done by intracutaneous running suture.

### Immobilisation

**Figure 1:**
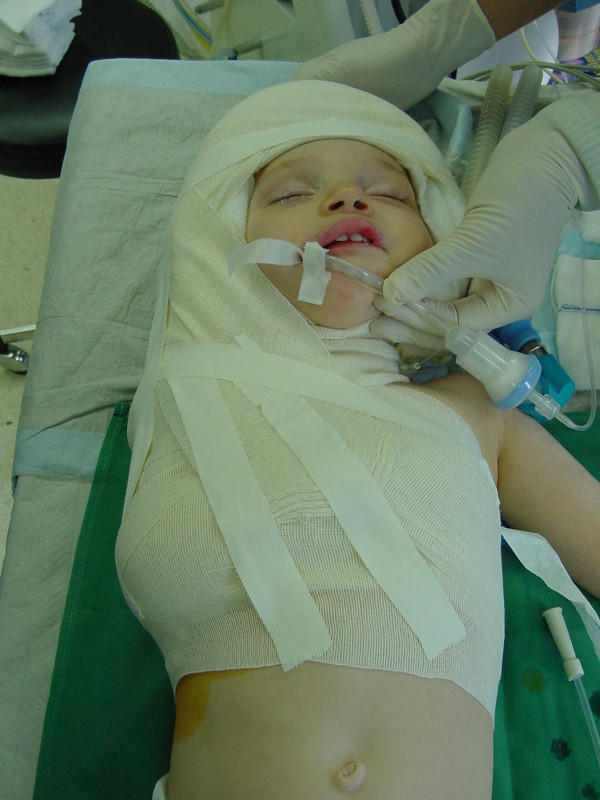
immobilisation helmet.

We actually immobilize the child in a custom – made plaster helmet (figure 1) for 3, in extreme extended lesions 4 weeks. The dressing is refreshed weekly, while holding the child to immobilize the affected arm and the neck.

After plaster removal, the arm is fixed for one additional week along the chest, the hand on the belly, to prevent any distraction on the retroclavicular coaptation.

### Follow-up

The plaster is removed in our Unit and children are seen every 6 months after surgery. Active movements are tested and contractures especially at the shoulder joint actively searched for.

Recovering muscle activities are recorded using BRMC and ROM scores.

### Possible errors

Some of them where real, some are hypothetic – as nobody can see again what happens to the reconstructed peripheral nerves.

Errors might be classified in various manners:

#### 1. intellectual

##### wrong timing

waiting too long before surgical decision:

This happens in the begin of experience... and is corrected once iterative surgical exposure of the brachial plexus shows how extended and real the lesions are. In all operated cases, the intraoperative status exceeded the preoperatively suggested extend of damage – so all the explorations were worth to be done!!

**Figure 2:**
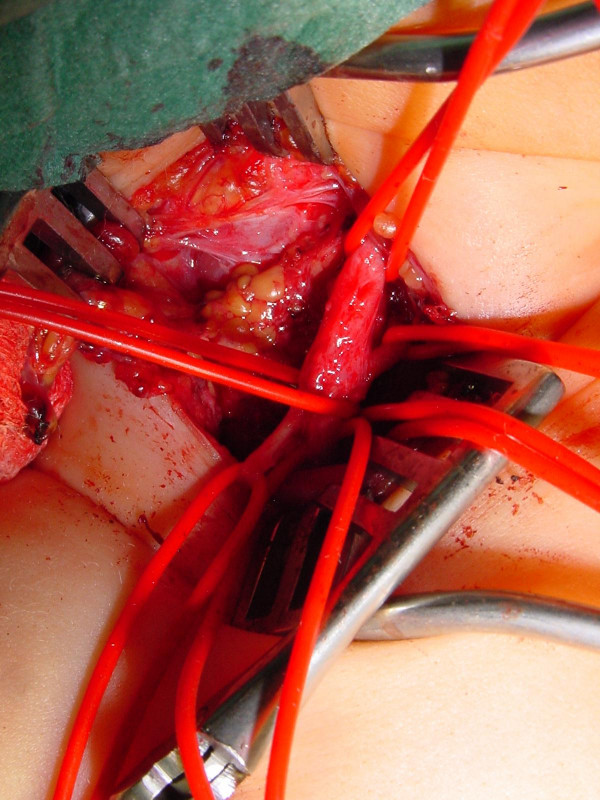
elective C6 avulsion: the lesion.

**Figure 3:**
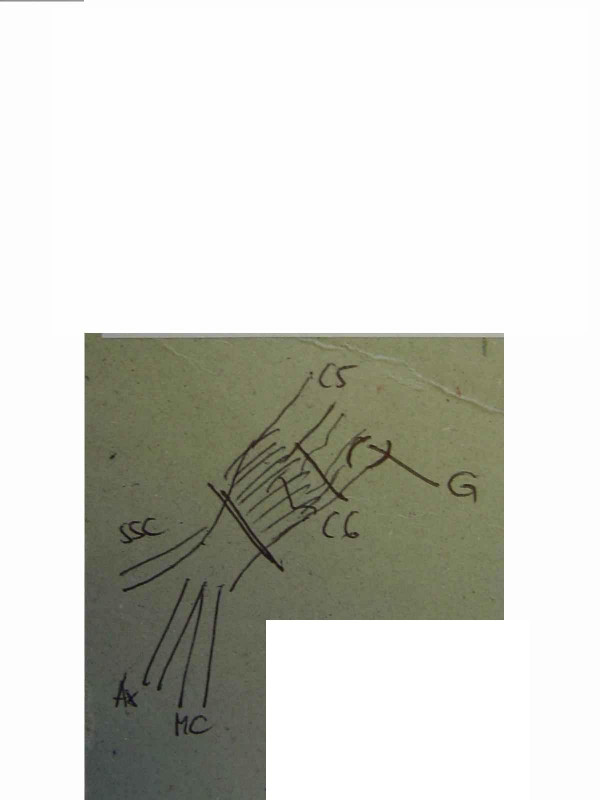
same case, drawing of the brachial plexus lesion.

**Figure 4:**
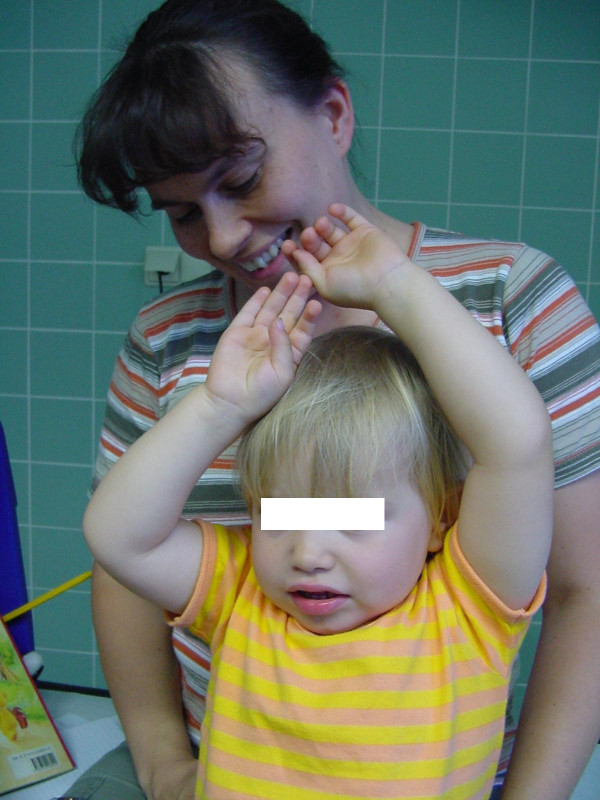
same case, after surgery (grafting of the upper trunk from root C5): recovery of shoulder abduction.

**Figure 5:**
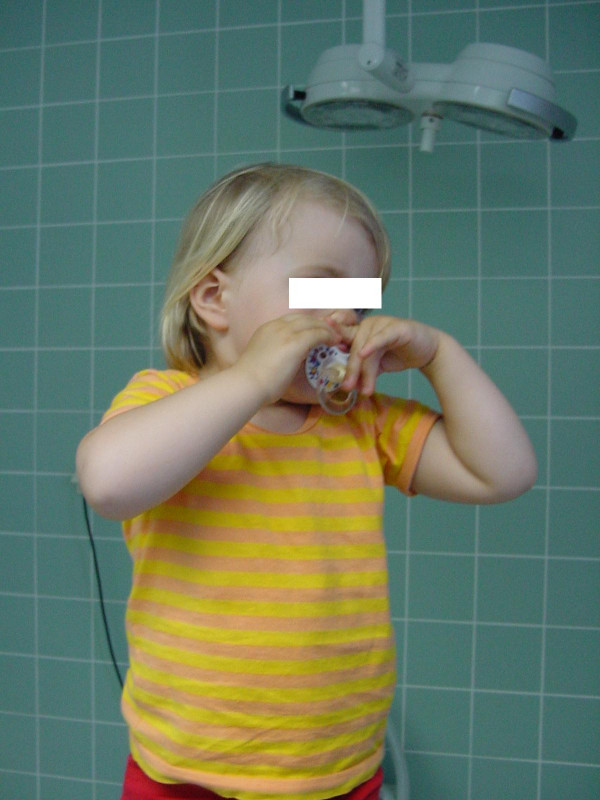
recovery of elbow flexion.

In one selected case, it took 6 months to decide: this girl had an upper lesion with a well recovering biceps, but a very bad shoulder... time and physiotherapy did not allow to show an improvement of abduction, and a single extraplexic neurotisation for the SSC nerve did not seem to be enough. Surgery showed an *avulsed root C6*; C5 was well conducting and was the donor for grafting to the whole upper trunk with a good recovery in shoulder and biceps (figures 2 to 5).

##### wrong strategy

there was a neurosurgical tradition in our country operating total obp lesions with the same reconstructive strategy like in adults, thus only upper roots where reconstructed and we heritated older children after total obp lesions and neurolysis or upper trunk grafts with totally flail hands.

**Figure 6:**
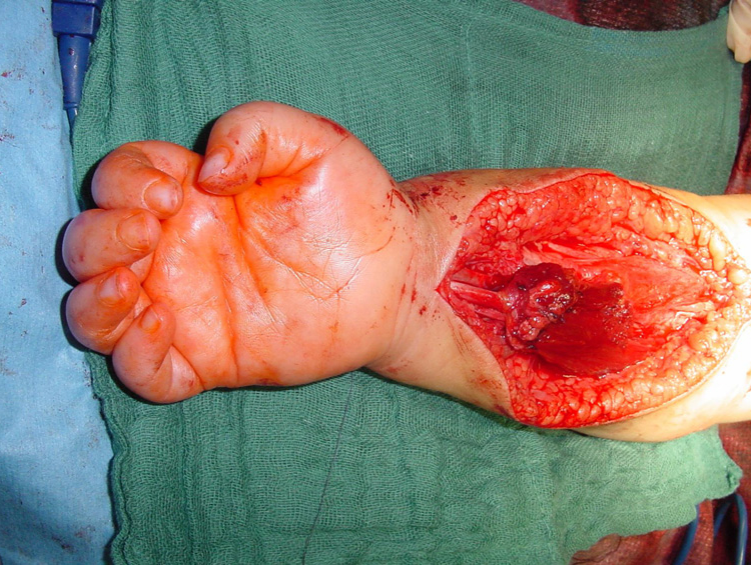
free functional gracilis transfer for finger flexors in a total lesion with flail hand.

2 of them have actually been reconstructed successfully by a free functional gracilis muscle transfer to the finger flexors (figure 6).

Neurolysis alone in severe lesions (non conducting neuromas, avulsed roots) is worthless [9]. We performed neurolysis alone in only 3 cases, where a scarring cuff surrounded fascicular trunk tissue. The recovery was good, but not as excellent (near normal) than expected.

#### 2. technical

**Tension on nerve sutures **or grafts must be avoided. In one „ideal" case of upper root reconstruction after excision of a short neuroma, direct coaptation and suture seemed possible and interesting while avoiding grafts.

Recovery was bad, a revision showed rupture of the coaptation site. Tension-free grafting led to a good functional recovery.

##### Glue or suture?

In ideal, tension free conditions, glue seems equal to suture. But the underface of the cable graft is difficult to be secured by glue- and glue does not prevent fascicle dislocation when an axial pull is exerted.

Sutures are more time-consuming, foreign material remains close to the fascicles, but slight tension might be compensated and the site of anastomosis might be visualised through the remaining operation.

##### Type and timing of immobilisation

10 days should be sufficient for a nerve coaptation – but what about the proximal and distal coaptation of a 4 cm graft, where the reinnervation cone reaches the distal junction only after 40 days (1 mm per day) – this period exceeds the usual immobilisation period of one month. What about passive stretching exercises on these shoulders and limbs, where the grafts have no extensive extra tissue?

##### Matching errors

Millesi [7] studied the nerve root topography in adults – should and could we apply this on tiny rootlets in 3 months old babies?

#### 3. compliance and reeducation

We still lack sound experience about the type and timing of physiotherapy. How strong parents should participate and put her children into reeducation patterns? How may this interfere with the conscious and motivated use of the operated arm in the long course?

As a conclusion, we ask several questions to the experts about technical details we would like to share:

1. How should the grafted nerves be enveloped, to assure better vascularisation and protection from scarring?

2. Could we decide about a unique immobilisation type and timing?

3. Which additional tools are helpful for good nerve regeneration: regular electrical stimulation? vitamine B complex? specific training regimens?

4. How should we glue? How extended must be the glue cone? Would it interfere with the regenerating fascicles jumping over the coaptation site?

5. Do we really need ongoing research on bioartificial nerve grafts, as we seldom lack grafts in children?

6. Could we focus on reinnervation markers, which would allow a postoperative monitoring – without re-operating ?

7. Are there factors promoting the muscular trophicity, while the nerve is regenerating and the muscle continues to atrophy?
